# Assessing the Quality of Physical Environments of Early Childhood Schools within the Cape Coast Metropolis in Ghana Using a Sequential Explanatory Mixed-Methods Design

**DOI:** 10.3390/ejihpe10040081

**Published:** 2020-12-18

**Authors:** Salome Amissah-Essel, John Elvis Hagan, Thomas Schack

**Affiliations:** 1 Department of Health, Physical Education, and Recreation, University of Cape Coast, P.O. Box 5007 Cape Coast, Ghana; salome.amissah-essel@ucc.edu.gh; 2 Neurocognition and Action-Biomechanics-Research Group, Faculty of Psychology and Sport Sciences, Bielefeld University, D-33501 Bielefeld, Germany; thomas.schack@uni-bielefeld.de

**Keywords:** center auspices, ECCD centers, modified CPERS, Cape Coast, Ghana, physical environment, quality

## Abstract

(1) Background: The last few decades have seen researchers giving considerable attention to the physical context of early childhood care and development (ECCD) centers because many of the underlying processes that link physical context are quite similar to psychosocial environmental factors regarding child development. However, research on the physical environments, and the employees’ understanding of the importance of physical environments, is often underestimated. The purpose of this study was to assess the quality of the physical environments of ECCD centers in the Cape Coast Metropolis, Ghana, and ascertain whether being a private or public center (center auspices) would be associated with the quality of its physical environment. A further inquiry into the educators’ understanding of the importance of physical environment on children’s developmental outcomes was made. (2) Methods: Using a sequential explanatory mixed-methods research design, all 160 ECCD centers in the Cape Coast Metropolis were assessed using a modified version of the Children’s Physical Environment Rating Scale (CPERS) and a semi-structured interview guide. (3) Results: Descriptive statistics indicated that more than half of the ECCD centers, 56%, rated “fair” on the quality of their physical environment. Although the locations and sites of these centers were of good quality, other physical environmental characteristics (i.e., “Planning of the Centre”, “Building as a Whole” and “Outdoor Space”) of ECCD centers were also rated to be fair. A Chi-square test showed that center auspices (i.e., being private or public) were not significantly associated with the quality of the physical environments of the centers [χ^2^_(2)_ = 2.490, *p* > 0.05], suggesting no significant difference between private and public ECCD centers in terms of the quality of their physical environment. A follow-up qualitative inquiry identified two themes as reasons why play yards in early years’ schools were not good: a ‘‘lack of funding” and “governmental support”. (4) Conclusions: Our findings suggest that the physical environments of ECCD centers are, to some extent, compromised. Stakeholders (e.g., Ghana Education Service, non-governmental/religious organizations, and private entrepreneurs) should help improve the quality of physical environments and also provide financial assistance for the provision of basic equipment (e.g., learning materials) for private and public ECCD centers in the Cape Coast Metropolis. Educators require in-service training to boost their in-depth understanding of the importance of physical environments on children’s developmental outcomes. Future studies could target children’s perceptions of their preschools’ physical environments as useful empirical information to help guide appropriate policy interventions.

## 1. Introduction

Child development literature reveals that the early years of a child’s education are essentially influential, and, across many societies of the world, attempts have been made towards investments by governments and other stakeholders to facilitate the development, learning opportunities, and healthy living for young children [1]. One significant index that plays a critical role in the attainment of these standards is the environment of the early childhood care and development (ECCD) centers, popularly referred to as preschools [2,3,4,5]. Berti et al. [6] indicate that a child’s early experiences with an excellent environment help form the architecture of the brain and set the foundation for the child’s lifelong success. Thus, positive outcomes are likely to occur if the child’s early experiences are positive. Conversely, undesirable outcomes are likely to happen if these experiences are negative. Some educational theorists and practitioners (e.g., Werner, Piaget, Montessori) have continually acknowledged the significance of physical space in an early learning environment and that a child’s physical environment is one of the key determinants towards his or her holistic development [7]. For example, the interactional-constructivist theory of child development and the environment places more emphasis on the physical environment by focusing on how the connections between the architectural and geographical environment and the social system separately and jointly influence how the child behaves [7]. According to Moore [8], educational environments well-endowed with refreshing stimuli offer diverse opportunities for exploration and testing. Maxwell [9] reiterated that the architectural design of the physical environment should boost a child’s sense of competence (i.e., an ability to discover the physical world with independence), generating opportunities for learning and play. Additionally, physical motor activities are essential to the health and general wellbeing of young children by promoting healthy cognitive development, weight gain, good cardiovascular condition [10], motor skill development, and psychosocial health [11] as well as lower adiposity, and increased bone density [12]. Since play and movement are important for brain development, preschool children should be exposed to activities that promote the development of fine motor and gross motor skills [13].

Scholarly reports over the years have established the susceptibility of children to negative health impacts of their degraded or unsafe environments [14]. Other studies have shown a correlation between ECCD center design and positive growth of children in preschool, suggesting that when the physical environment is comfortable, it influences children’s play behavior, which leads to better learning [15,16]. Similarly, the role the physical environment plays on other factors determines the quality of teaching and learning, that is, the educator’s effectiveness as well as the child’s performance and overall growth [3,9,17,18,19,20]. In contrast, exposure to poor quality physical conditions is also associated with psychosocial conditions [21]. For example, preschools with high-quality physical environments have children exhibiting fewer anxious and distress behaviors [22]. Similarly, good quality physical environments in ECCD centers have been found to be helpful for little children from disadvantaged backgrounds (i.e., poor homes) as they are provided with opportunities and experiences not given in their houses [23].

Other extant research literature indicates that three explicit physical environmental design parameters are considered most essential in early childhood learning: spaces that boost exploration, independence, and development (i.e., a child’s sense of self and willingness to play); spatial quality (e.g., color, light, noise, and materials); and the amalgamation of outdoor and indoor environments [2,7,9]. According to Curtis and Carter [24], a very thoughtful and appropriate architectural design of physical space can foster a child’s quest for exploration, learning by means of play, peer interaction, and self-confidence improvement and social skills. A suitably designed space could improve a child’s sense of competence and offer a serene place that provides maximum security and comfort. These development benchmarks would help provide an identity and a sense of self-worth through exploration and play for these young children [9,25]. Other research evidence suggests that children benefit from their overall well-being as well as physical health when their preschools provide substantial opportunities for outdoor play and have contact with nature [26,27]. Bagot [28] further stated that children who attend more “natural” daycare centers display better motor skills abilities, increase their attentional capacity, and have fewer sick days. Additionally, spending some time in the sun during outdoor play improves children’s health and minimizes the risk of sick building syndrome, which is usually linked with inadequate access to natural daylight and fresh air in indoor settings [29].

The connection between physical environment and early childhood developmental outcomes in the current study could be considered from a theoretical perspective, using the constructivist approach. This premise is based on the idea that an understanding and knowledge of the environment in which people live is co-constructed through vicarious experiences of the immediate environment and reflections on those experiences [30,31]. According to this perspective, perception of space is considered very important. For instance, the physical features of spaces impact the perception and representation of reality; they define the context in which people can act and live [31]. From this theoretical standpoint, specifically accounting for the physical features of early childhood environments and better understanding educators’ perspective on children’s developmental outcomes may provide useful emerging themes through meanings and behaviors of individuals who inhabit these contexts [32,33].

To date, many countries in sub-Saharan Africa depend primarily on public–private early childhood care and education through collaborations with non-governmental organizations (NGOs) for community-based initiatives. Early childhood care and education services in Ghana, as in other sub-Saharan states, are provided by the state and private institutions such as NGOs, religious organizations, communities, and commercially-oriented private entrepreneurs with varied motives for their participation in early childhood education [34,35]. Although some of these ECCD centers have relatively good environments (i.e., adequate and appropriate play and other physical facilities, clean and hygienic sanitary facilities), quite a number lack the appropriate environment to promote effective teaching and learning. 

Ghana has made great progress in early childhood education, with evidence showing significant increases in enrolment in ECCD centers in the country, which have exceeded the goal for preschool enrolment [1]. Despite the Ghana Education Act, 2008 [36] legislation providing legal directives and policies supportive of the restructuring and transformation of the physical environment, as well as the educational facilities of preschools, earliest years’ schools have seen no structural transformation. These centers still operate in unchanged and undesirable physical environments in the midst of their huge enrolment sizes. A scenario that eludes one of the stated objectives of the Ghana Education Act, 2008 is “to redefine and augment education and support services that are responsive to the needs of all children, within the context of universal design and child-friendly schools, and overall, to increase participation and educational access for children, including those with special needs”. To accomplish this educational policy goal, the physical architectural designs and environments of existing schools ought to be reformed or adapted, while also guaranteeing that all new school physical designs and constructions enhance opportunities for all children.

Given that a large body of research exists on how different physical design features influence child development and behavior, it is surprising that scholarly information on early childhood educators’ understanding of the physical designs of their early childhood learning centers within the Ghanaian context is undocumented. Additionally, research on the extent to which specific physical environment characteristics of ECCD centers are identified and assessed to promote learning in the country is limited. To date, only a few studies have investigated issues related to the physical environments of ECCD Centers in Ghana. For example, Bidwell, Watine, and Perry [37] explored ECCD programs in peri-urban settings in Africa and found that fundamental structures, such as toilet facilities and playing fields, enclosed spaces around the school, and electricity mostly existed in preschools in Soweto (South Africa) and Ashaiman (Accra, Ghana). Bidwell and partners noted that these infrastructures were lacking, in substantial proportion, in preschools in Mukuru, Nairobi, Kenya. Similarly, evaluating preschool programs of selected schools for the deaf in chosen Ghanaian cities (i.e., Cape Coast, Sekondi, Kibi, Koforidua, and Mampong-Akuapem), Larbi [38] found that most preschools for the hearing impaired had environments and indoor, as well as outdoor, learning spaces that were conducive to learning and development. However, the playgrounds were not spacious enough for the preschoolers, and most classrooms were not spacious enough to accommodate the children and their indoor equipment. Summarily, these few studies did not provide an in-depth assessment of the qualitative impressions of school educators (i.e., employees’ understanding) on how physical environment indices might promote learning in these educational institutions. To the best of our knowledge, no research has accounted for employees’ understanding of how ECCD centers’ architectural designs might promote learning. Further, given governmental support for public schools compared with private ones across all levels of education in Ghana, examining whether center auspice would be connected to the quality of the physical environments of ECCD centers could provide useful information for policy realignment. This comparative assessment has been ignored by previous studies. Besides, even though the impact of physical environment on children’s holistic development appears to be critical at all educational stages [5], educators’ (e.g., employees, heads, coordinators) understanding about their working spaces regarding the children’s physical environments are vital. Therefore, this current study assessed the quality of physical environments of early childhood schools within the Cape Coast Metropolis, Ghana by employing a sequential explanatory mixed-method approach. Additionally, whether ECCD center auspices (i.e., being private or public) would be associated with the quality of the physical environment often associated with overall child development was examined. A further inquiry was made on educators’ in-depth understanding of the importance of how ECCD physical environment features would be associated with overall children’s developmental outcomes. 

It was hypothesized that physical characteristics of ECCD centers incorporated as part of the physical environment of the ECCD centers would rate higher in quality, according to the standard ratings of the adapted Children’s Physical Environment Rating Scale (CPERS) inventory. Based on more governmental support (e.g., infrastructure, financial) for public schools than for their private counterparts in Ghana, it was anticipated that public ECCD centers would rate higher than private ECCD centers on the CPERS physical environment indicators. Given that educators’ views are central to children’s developmental outcomes, additional hypotheses were drawn from the quantitative results that recorded the least mean scores on the selected CPERS indicators. It was further hypothesized that ECCD coordinators would demonstrate an adequate understanding of the value of physical environment and relate the values to young children’s developmental outcomes. ECCD centers in this study included all the institutional service centers that take care of children from birth until school age (i.e., facilities that take care of children from 0 to 6 years old). These facilities were primarily day-care and childcare centers, nursery schools, preschools, and kindergartens in the Cape Coast Metropolis. The physical environment in the current study referred to the overall design of a center, covering features such as size, density, privacy, well-defined activity settings, modified open-plan space, a variety of technical design attributes, as well as outdoor play spaces, which are linked to the emotional, social, and cognitive development of children [6].

## 2. Materials and Methods

### 2.1. Study Design

The study was conducted using a sequential explanatory mixed-methods approach, where the quantitative part precedes the qualitative component, a design usually noted for exploring new phenomena [39,40,41]. According to Hancock [42], this research design is very essential towards the attainment of both in-depth experiences and general realities that frequently reconstruct social stratification along numerous loci of marginalization. Given that there is sparse empirical evidence on the quality of physical environments of the early years schools in the Cape Coast Metropolis, Ghana, using the sequential exploratory mixed-methods approach serves as the most preferred design for this inquiry. Ethical approval for this study was obtained from the Institutional Research and Ethics Committee, University of Cape Coast, Ghana (UCCIRB/CES/2016/01). Permission to conduct the study was initially obtained from the Central Regional Education Directorate of the Ghana Education Service, Cape Coast Metropolis.

### 2.2. Study Area

The study was conducted in the Cape Coast Metropolis, the capital of the Cape Coast Metropolitan District and Central Region of Ghana. Cape Coast is situated in the south of Ghana, on the Gulf of Guinea, and has a population of 169,894 people [43]. This city is about 146 km away from the national capital, Accra. The European merchants introduced castle schools within its catchment areas along the coast. The formation of these schools gave birth to the introduction of formal education in the country, including Cape Coast. Currently, there are 160 early childhood centers in the Cape Coast Metropolis, with 101 being private and 59 public. Primarily, Kindergarten Education has a two-year preschool program offered mostly by communities and private organizations, with technical support from the government (i.e., Ghana Education Service). The preschool program is offered to help children learn to communicate, play, and interact with others appropriately [44]. Educators at the preschool centers provide various learning materials and activities to motivate children to learn their local and English languages through music, art, physical activities, and social behaviors.

### 2.3. Quantitative Phase

#### ECCD Center Inclusion

The current study used a census approach for sampling all registered ECCD centers in the Cape Coast Metropolis that were in existence. The Ghana Education Management Information System (GEMIS, [45]) records had initially indicated 163 ECCD centers in the Metropolis. However, only 160 ECCD centers were in existence during the field data collection. The other three centers had either relocated or closed down. The characteristics of the ECCD centers chosen for the study are presented in Table 1.

### 2.4. Instrumentation

A modified version of the Children’s Physical Environment Rating Scale” (CPERS, [7]) was used for this study. The original CPERS questionnaire has 124 items organized into 4 parts of 14 subscales, with each subscale having different items. Part A focuses on the overall planning of the center (e.g., building size of the center: length, breadth); Part B looks at the environmental quality of the building, overall organization, image, and circulation (e.g., children can see some indoor activity areas from outside before entering the center); Part C assesses each module (classroom) and spaces where children spend the majority of their time (e.g., children activity areas are partially enclosed to provide protection from visual and noise distractions); and Part D evaluates the outdoor activity area around the building, and surrounding conditions (e.g., the total area useable outdoor play yards: length, breadth). 

Based on the rationale of this study, that is, to assess the quality of physical environments in relation to developmental outcomes of children, the CPERS was modified by excluding the subscales assessing the “Quiet Activity Area” and “Messy Activity Area”. Under the subscale for the “Physical Activity Area”, the items measuring the “Music Area” and “Dramatic/Fantasy Play Area” were removed. Additionally, the items measuring the Arts and Crafts Studio, Water Play Area, and Science and Nature Area were also removed. These specific areas and provisions are usually not provided as part of a preschool’s physical environmental design in the Ghanaian context, and therefore might be redundant information or not relevant to the instrument. Therefore, the modified CPERS used in the current study constituted 84 items, categorized into 4 parts of 12 subscales. To ascertain the validity (i.e., construct and content) of the modified CPERS, a group of three experts (i.e., professors) considered the overall instrument and its themes. These experts considered the validity of the “items” under each subscale with particular reference to the general Ghanaian school set-up. The researchers assessed the building of ECCD centers by deciding on how well each center satisfied each item on the subscales anchored on a five-point linear-numeric scale, starting from “Not Met” (score of 0) to “Fully Met” (score of 4). After completion, each subscale score was calculated using a mean of the items on a particular subscale. The total score for an ECCD center was then calculated based on a grand mean of all the subscale scores. Previous studies have reported internal reliability (Cronbach alpha) values ranging from 0.53 to 0.96 on all the CPERS subscales [7,46,47]. Calculated Cronbach coefficient alpha values in the current study ranged from 0.63 to 0.97 on all the chosen modified CPERS subscales (see Table 2). These coefficient figures are acceptable and consistent with previous research [7,46,47,48]. 

### 2.5. Procedure

The researchers obtained permission letters from the Cape Coast Metropolitan Education Directorate and the Social Welfare Department. The letters of intent were sent to all the centers. Standard ethical practice and interview procedures were followed after approval by the institutional review board (IRB), University of Cape Coast, Ghana. Informed consent was obtained from each ECCD center through the institutional head to participate in the study, which involved two research protocols: qualitative interviews with center heads and quantitative analysis (i.e., measurement) of each center’s physical environment. The researchers booked an appointment with the centers and discussed the rationale of the study, that is, to assess the physical environment (i.e., architecture and the built environment of the center) and that the study was not looking at curriculum, staffing, or the children. Two research assistants were used in the physical measurements of the classrooms (length and breadth), the play yard, and the center building, after they were trained on how to read and use a measuring tape. The actual assessment of the physical environment of all the centers was done by one of the researchers (i.e., principal author), and a hired architectural expert, together with the two trained assistants, through live visits to classrooms at the ECCD centers. Specific guidance from the expert rater, whose readings and codes were presumed to be acceptable, was followed throughout the data collection period [49]. To avoid disrupting the ECCD centers’ academic work, the data collection exercise was staggered, with a duration of three months.

### 2.6. Data Analysis

Descriptive statistics (means, frequencies, percentages) between the physical parameters under study variables were computed. To get the overall quality of the physical environment, the total score of the modified CPERS was calculated by finding the mean score for all 12 subscales, and the final scores were grouped as follows: 0.00–1.00 = “Poor”, 1.01–2.00 = “Fair”, 2.01–3.00 = “Good” and 3.01–4.00 = “Excellent”. Pearson’s Chi-square test of independence was also performed to determine the extent to which center auspice (i.e., private or public) was significantly associated with the quality of the physical environments of the ECCD centers. During the Chi-square test, it was found that the “Poor” environment category had one cell indicating zero cases. Therefore, the category of “Poor” could not be added for the analysis, leaving three categories under the quality of the physical environment variable. 

## 3. Results

### 3.1. The Quality of the Physical Environment of ECCD Centers in the Cape Coast Metropolis

The results indicate that more than half of the ECCD centers, 56% (N = 89) rated “Fair” on the quality of their physical environments, while only 14% (N = 23) of the ECCD centers have an “Excellent” rating on the quality of their physical environments (see Table 3). Other results show that the majority of the ECCD centers, 56% (N = 89) scored fairly on three parts of the physical environment: “Planning of the Centre”, “Building as a Whole”, and “Outdoor Space” (see Table 4). 

To further explore the overall performance of the centers on the rating of the physical environment, Table 4 indicates that the centers scored high on ‘Location and Site’ (Mean (M) = 2.95, Standard Deviation (SD) = 0.58). However, consistent with the results shown in Table 3 is the evidence from Table 4 showing the majority of the mean scores ranging from 1.01–2.00 (“Fair” rating). Specifically, “Planning of the Centre” (Centre Size and Modules (M = 1.59, SD = 0.53), “Building as a Whole” (Common Core of Shared Facilities” (M = 2.02, SD = 1.14), and “Outdoor Space” (Play Yard: Functional Needs (M = 1.90, SD = 0.65) and Play Yard: Developmental Needs (M = 1.84, SD = 0.84)) were realized. The overall modified CPERS Total Score indicated a “Good” rating (M = 2.23, SD = 0.86).

### 3.2. Chi-Square Results on Centre Auspices’ Association on the Quality of Physical Environments of ECCD Centers

Table 5 shows the results of the test for association between center auspices (private or public) and the quality of their physical environments (Fair, Good, and Excellent). Centre auspices were not significantly associated with the quality of physical environment of the centers (χ^2^_(2)_ = 2.490, *p* > 0.05), suggesting no significant difference between private and public ECCD centers in terms of the quality of their physical environments. 

### 3.3. Qualitative Phase

#### 3.3.1. Participants’ Inclusion Criteria

Eight ECCD center heads and coordinators were purposively sampled for the follow-up interviews. The inclusion criteria were solely based on the educators’ ability to proficiently speak English and the local Fante language of the metropolis, who have been at the facility for at least five consecutive years, and were willing to be interviewed.

#### 3.3.2. Need for Follow-Up Explanations

From the results of the overall performance of the centers on the rating of the physical environments, as shown in Table 4, it was found that the centers had the lowest scores in “Planning” (Centre Size and Modules (M = 1.59, SD= 0.53)) and “Outdoor Space” (Play Yard: Functional Needs (M = 1.90, SD = 0.65) and Play Yard: Developmental Needs (M = 1.84, SD = 0.84)). The purpose of the follow-up section of this study was to ascertain the detailed reasons why ECCD centers did not perform well in the physical environment aspects that looked at “Play Yard”, meeting both the functional and developmental needs of the children at the centers. These specific two indicators were selected because the educators/coordinators have no direct role in the establishment of the ECCD centers and the modules used are solely determined by supervising governmental institutions. Another reason is the “Centre size and Modules” result is not too surprising because, over the years, Ghana has seen a rise in preschool enrolment and has even exceeded the nation’s target of both gross and net enrolment ratios, as of the 2013/14 academic year [1]. Hence, there was no follow-up interview on this subject matter, and the following two questions were posed to guide the semi-structured interview phase:

- Do ECCD center heads/coordinators recognize the importance and support the provision of outdoor spaces for children to play?
- Why do play yards in ECCD centers not meet both the functional and developmental needs of the children?

Table 6 presents the demographic characteristics of the eight selected participants interviewed in the study.

### 3.4. Measures

#### 3.4.1. Qualitative Interviews

The semi-structured face-to-face interviews were done to cover the following areas: the extent to which the physical environment had an influence on the ECCD center, the relative importance of the physical environment on a child’s development, center characteristics or features constituting quality physical environment, as well as the design expectations of ECCD centers. Other preliminary questions focused on the demographic characteristics of the heads/coordinators, such as their age, gender, experience, qualifications, as well as the type of ECCD center they work in.

#### 3.4.2. Analysis 

Transcripts were coded and analyzed using both manual and computer-assisted qualitative data analysis software (CAQDAS), referred to as NVivo 11 Plus. For quality checks purposes, expert and independent coders were used for the template analysis coding portion of the collected data for later subsequent discussion. Preliminary inter-coder reliability of the coding was determined using the Holsti’s coefficient [50] because the chance that two coders might use the same quote of a participant by chance as a reason why ECCD centers did not have enough equipment was deemed negligible. The Holsti formula is the following:

C.R. = 2M/N_1_ + N_2_ (1)

where M is the number of coding decisions on which the two judges were in agreement, whereas N_1_ and N_2_ referred to the number of coding decisions made by Judges 1 and 2, respectively (p. 140). In the current study, two coders coded all eight cases (quotes), agreed on seven cases, and disagreed on one case. The cause of the disagreement was that one of the coders coded a participant’s quote as a funding reason, while the other coder coded it as a government support reason. Therefore, the inter-coder reliability was C.R. = 2(7)/8 + 8, which was equal to 0.875, a figure considered very reliable [51].

The main analysis involved a thematic approach identifying key categories, themes, and patterns. Following the recommendation of Saldana [52], four key iterative steps were followed: mechanics (transcription), data immersion (i.e., reading and re-reading transcripts), which involved using phrases or sentences to describe or capture the meaning of an aspect of the data, generating initial codes in vivo (i.e., initial pattern recognition using participants own words), and theming (i.e., categorizing key themes and sub-themes through the identification of meaningful categories) based on the code frequencies [53]. Emerging themes were revised and refined into main themes and sub-themes, with the results specifically capturing various excerpts from the raw data that had the exact words of study participants, so that readers can assess the different thematic constructions from the findings.

### 3.5. Results and Explanation of Themes

#### 3.5.1. Participants’ Views on the Importance of Outdoor Spaces

All the ECCD heads and coordinators responded positively that having a space for children to play was very important. Explaining why they responded positively to the question, three themes were identified and are presented in Table 7 (a, b) shows some of the extracted responses under each of the themes identified.

#### 3.5.2. Why Play Yards do not Meet the Functional and Developmental Needs of Children

Two themes were established on participants’ responses to the question “Why do play yards not meet the developmental needs of children?” Table 8 (a) shows the themes identified, whereas Table 8 (b) presents extracted responses concerning the themes identified.

## 4. Discussion

One key indicator of the quality of early childhood education programs is the physical environment within which education and care are provided [5]. The purpose of this current study was to assess the quality of the physical environment of early childhood care and development (ECCD) centers in the Cape Coast Metropolis, Ghana, and ascertain whether being a private or public ECCD center (center auspice) would be associated with the quality of its physical environment. Additionally, a further inquiry on educators’ in-depth understanding of the importance of how ECCD physical environment features would be associated with overall children’s developmental outcomes. 

### 4.1. Quality of the Physical Environment of ECCD Centers

The hypothesis related to the quality of physical environments, as measured by the modified CPERS indicators, was partly supported. Our findings suggest that the overall physical environment of ECCD centers in the Cape Coast Metropolis is of a fair quality, a result that echoes similar research findings [5,37,54,55]. The majority of ECCD centers scored fairly on all four main parts of the physical environment (i.e., “Planning of the Centre”, “Building as a Whole”, Indoor Spaces”, and “Outdoor Spaces”). Consistent with the overall quality ratings of the physical environment, the results for the 12 physical environment indicators revealed that the ECCD centers rated fairly on half of the indicators. Previous studies have established that the quality of the physical environment in ECCD centers in relation to the building, open spaces, and the quality of outdoor play spaces are associated with children’s cognitive, social, and emotional development [21]. Barbour [56] noted that the design of play spaces is connected with children’s development by either enabling or hindering specific sets of behaviors. According to some researchers [3,5], the effectiveness of the preschool program is often hampered, non-fetching, and uncomfortable because of inadequately designed and disorganized physical environments. For instance, preschools with high-quality open and outdoor spaces have children exhibiting fewer anxious and distress behaviors [22], which subsequently influence their play behaviors towards learning [16]. Therefore, a well-organized physical environment (e.g., building, indoor, and outdoor spaces) is likely to contribute decisively to children’s adjustment in school and foster positive interactions between children and their teachers, thus promoting children’s moods and behaviors, as well as the quality of the educators’ work [9,17]. Other research on the quality of physical environment has shown a stronger positive relationship with children’s academic and literacy skills [57]. 

One area of concern that needs considerable attention is “Centre size and Modules” because current results imply that most centers in the Cape Coast Metropolis could have more children at the centers than their current capacities. This finding calls for the expansion of existing physical structures of the ECCD centers.

### 4.2. Variations on ECCD Centre Auspices (i.e., Being Public or Private) on the Physical Environment

The quality of physical environments across center auspices (i.e., being private or public) was compared on the premise that many countries in sub-Saharan Africa, including Ghana, depend primarily on public–private early childhood care and education through collaborations with non-governmental and religious organizations, as well as private entrepreneurs. The formulated hypothesis was not supported because current findings revealed no significant difference between private and public ECCD centers in terms of the quality of their physical environments. The results from the profile of the 12 physical environment indicators suggest that the most obvious strength of ECCD centers within the Cape Coast Metropolis appears to be “Location and Site”. This finding indicates that the dimensions of the outdoor spaces in the physical environments, together with the locations and sites of ECCD centers in the Cape Coast Metropolis, are good. The plausible reason is that the Cape Coast Metropolis is not a heavy industrial area. Rather, it enjoys a moderate business climate, which includes petty trading, crafts and other manufacturing, institutional workers, professionals (public servants—largely teachers), agriculture (farming and fishing), and fish processing [58]. Therefore, the location and sites of most of the ECCD centers in the Metropolis do not primarily expose children to notable harmful pollutants, especially from neighboring industrial facilities or contamination from past industrial use of land. This current finding is good and worth noting. Various stakeholders should make all efforts to maintain the outdoor environmental quality, especially in areas where these ECCD centers are situated. Evans [21] reiterated that the neighborhood set-up for ECCD centers is critical because some physical environmental conditions may pose potential developmental challenges for children. For example, some negative consequences (e.g., attention problems and movement-related challenges) have been linked to neighborhoods characterized by economically deprived conditions after controlling for individuals’ socio-economic statuses [59]. 

### 4.3. ECCD Centre Heads/Coordinators’ Understanding on the Importance of Outdoor Spaces and Play Yards for Children’s Functional and Developmental Needs at ECCD Centers 

The formulated hypothesis that ECCD coordinators would demonstrate an adequate understanding of the value of outdoor space and relate the values to young children’s functional and developmental outcomes was partially supported. Follow-up interviews with the various heads/coordinators of the ECCD centers in the Cape Coast Metropolis partly acknowledged the importance of outdoor spaces towards children’s learning experiences. 

Substantiating previous research [7,25,60], the importance of specific features of the physical environment, such as outdoor spaces, was seen by heads/coordinators as a factor in children’s enjoyment and learning at ECCD centers. Analytically, whereas these interviewed heads/coordinators valued adequately designed open or outdoor spaces, they gave abstract responses to the concept of physical spaces and could not clearly express which specific elements (e.g., scale, form, organization) influence children’s development and learning [2]. Importantly, it is crucial that ECCD center heads and coordinators demonstrate a thorough understanding of how well a center space, designed with tangible educational materials and not merely the availability of ideal space, could provide better communication and social interactions towards enhanced learning for children. Heads and coordinators’ ability to vividly describe the ideal physical environment could provide architectural designers with a significant understanding of design prioritization for the ECCD centers [2]. For instance, ECCD personnel’s understanding of ‘modified open-planned space’ with small and large play areas open enough for children to see out, related to important educational messages (i.e., contents), could change specific interactions between teachers, children, and the physical environment. These parameters may offer a sense of confidentiality in children’s play, safeguard them from noise and distraction, as well as facilitate their attention and learning retention [3,5,6]. The design implication for this proposition is that a greater value would be placed on general indoor and outdoor orientation when first establishing an ECCD center, regardless of whether it is being established as a private or public educational set-up. 

Additionally, the interviewed educators indicated that time spent in outdoor areas is key to promoting effective learning and development among preschool children [61]. The heads also indicated that playing outdoors helps with the upkeep of the children. This finding corroborates other research that spending some time in the sun during outdoor play can help improve children’s health [35]. Additionally, ECCD center heads/coordinators also indicated that children need to play so that they do not become dull. When children are involved with play in an indoor or outdoor environment, their motor skills and abilities are significantly improved. Researchers [62] on physical education and movement science highlight the significant association between the fundamental period of preschool children and their development of motor skills. The Council on Physical Education for Children [63] emphasize that preschoolers, whose physical environment promotes physical activities, enhance their positive attitudes toward health and fitness. Other research has shown that the lack of physical activity in childhood is associated with sedentary behavior in adolescence and later adult life [64], often related to various health risk conditions (e.g., obesity, cardiovascular diseases) in adulthood [65]. Therefore, indoor and outdoor spaces that foster preschoolers’ consistent involvement in physical activities during their early years may be the first step towards developing their psychomotor skills that positively contribute to a healthy lifestyle in later life [62].

Other areas of weakness shown by the profile of the 12 physical environment indicators were the “Play Yards: Functional Needs and “Developmental Needs”. A follow-up interview with the center coordinators revealed that the centers were characterized by inadequacies of basic equipment (e.g., playing and educational materials) appropriate for children and playgrounds, due to a lack of adequate financial support. The educators showed appreciable understanding of why play yards were not meeting both the functional and developmental needs of children in the Cape Coast Metropolis, a finding consistent with other research [66]. Although basic equipment has a significant influence on children’s development [18], there seems to inadequate attention by supervising authorities on the provision of these learning materials for ECCD centers in the city. According to Woodhead [67], most preschool yards across many parts of the world are just flat, hard, and open surfaces that reflect a traditional belief that children’s learning only takes place in the classroom—a scenario that mirrors play yards of the ECCD centers in the Cape Coast Metropolis. 

### 4.4. Limitations

The current findings should be interpreted in light of some limitations. This study was restricted only to the Cape Coast Metropolis in the Central Region of Ghana, one of the numerous Metropolises in the country. Therefore, the current findings limit generalizability to other geographical areas of the region and/or other parts of the country. A larger and more diverse sample group is required to fully appreciate the current and future role of the physical environment in the Ghanaian context and undertake explicit observations and assessments of other areas of physical environment not captured in the current study. Additionally, children’s data (e.g., growth pattern, cognitive, physical, and intellectual development) and other discriminant variables (e.g., socioeconomic background of the neighborhood, years of center creation) were not measured in the current study, hence, causality cannot be ascribed to current findings. Future studies could consider which specific physical environmental features of ECCD centers could predict children’s intellectual and cognitive development. Further, given that CPERS could be used by different people (e.g., researchers, school staff, educators, and architects) who might differ in their knowledge and experience about early childhood programs, the use of test-retest and/ or inter-rater reliability, and possibly testing the psychometric properties of modified versions of CPERS would be appropriate. However, the number of ECCD centers (N = 160) in the current study and time constraints made using these reliability approaches practically impossible. Though the modified CPERS was validated by experts, the reported scores from the modified instrument should be noted with caution.

Despite our relatively small and homogeneous sample of eight educators (heads/coordinators), the use of a sequential exploratory design justified a full examination of the study variables. Gathering interview or focus group data to show emerging patterns or trends related to the physical environments of ECCD centers in early childhood education as a follow-up to a survey could enhance the correctness and precision of the quantitative findings [68]. This research design, according to Cabrera, could provide openings to assess the validity of personal perceptions on how the physical environment could help promote early childhood development through personal experiences that are considered very essential.

### 4.5. Practical Implications

The first early childhood in-school experiences predict later school accomplishment. Hence, the environment for children ought to offer a productive early school transition period, considered a ‘‘sensitive period’’ for later school success [69]. This transition period should promote a developmental pathway for the child through his or her social and physical environment that boosts new opportunities for personal growth [70]. The trajectory for development at this critical stage may also show an inverse association with the direction of the child’s educational career. Consequently, the reasons that may impact this trajectory permit great consideration [71].

Immediately, when children enroll in ECCD schools, their protection and wellbeing, and access to buildings and teaching as well as recreational areas should be assured [72,73]. When the comfort of every child is guaranteed, effective learning will improve. Hence, the educational value of the physical environment should not be underestimated in terms of its organization and the experiences it provides. The interactions children have with the physical environment build a great bond with their teachers, promote their health, and boost overall mood and behaviors that ultimately build their development (e.g., psycho-motor), learning outcomes (e.g., literacy), and overall wellbeing (e.g., health [9,17]).

## 5. Conclusions

Summarily, the quality of ECCD centers in the Cape Coast Metropolis is not the best. The current study revealed that even though the locations and sites of early childhood schools in the Cape Coast Metropolis were of good quality, the overall assessment indicated that the physical environments (e.g., “planning of the centre”, “building as a whole”, indoor spaces”, and “outdoor spaces”) of these ECCD centers were of average standard. There was no significant difference between center auspices and the physical environment of ECCD centers in the Cape Coast Metropolis. Play yards of these schools do not meet the functional and developmental needs of the children. The relative importance of ECCD centers providing children with outdoor and play yard spaces was also acknowledged by heads/coordinators of these early childhood centers. 

Based on the current findings, locations and sites of the ECCD centers should be maintained by supervising authorities (e.g., Ghana Education Service). Both private and public preschool establishments in the Metropolis should be supported financially for the provision of basic equipment for these centers by governments, organizations, and private entrepreneurs. The establishing institutions should help improve the quality of the physical environments of these early childhood centers. The supervising institution (e.g., Ghana Education Service) could also provide in-service training for educators to boost their in-depth understanding of how physical environmental characteristics can effectively and developmentally promote effective teaching as well as learning. For a detailed understanding of the multidimensional perspective of the quality of physical environments and learning/developmental outcomes, future longitudinal and/or interventional studies could target which specific physical environmental features would elicit children’s developmental outcomes across specific age cohorts in Ghana. Further exploration of children’s perceptions of their preschools’ physical environments could provide useful empirical information for appropriate policy interventions.

## Figures and Tables

**Table 1:** Frequency Distribution of Early Childhood Care and Development (ECCD) centers in Cape Coast Metropolis by Auspices.

Early Childhood Care and Development (ECCD) Center Characteristics	Frequency	Percentage
Public	59	37
Private	101	63
Total	160	100

**Table 2:** Means (M) and Standard Deviations (SD) of Modified CPERS Scores with Subscale Reliability Values.

Sub-Parts of the Physical Environment	M	SD	Cronbach Alpha
**Planning**			
(1) Centre Size and Modules	1.59	0.53	0.84
Building as a Whole			
(2) Image and Scale	2.53	0.93	0.88
(3) Circulation	2.49	0.77	0.93
(4) Common Core of Shared Facilities	2.02	1.14	0.96
(5) Indoor Environmental Quality	2.28	1.12	0.96
(6) Safety and Security	2.60	0.76	0.89
**Indoor Activity Spaces**			
(7) Modified Open-Plan Space	2.53	0.96	0.94
(8) Home Bases	2.06	1.14	0.97
(9) Physical Activity Areas	2.08	0.91	0.96
**Outdoor Spaces**			
(10) Play Yards: Functional Needs	1.90	0.65	0.63
(11) Play Yards: Developmental Needs	1.84	0.84	0.91
(12) Location and Site	2.95	0.58	0.81
**CPERS**			
CPERS Total Mean Score	2.24	0.86	
Modified CPERS	84 items	-	0.63–0.97
Original CPERS	124 items	-	0.53–0.96

Note: N = 160

**Table 3:** Quality of the Physical Environment in ECCD Centers in the Cape Coast Metropolis.

Quality	Mean Score	Frequency	Percentage
Poor	0.00–1.00	7	4.4
Fair	1.01–2.00	89	55.6
Good	2.01–3.00	41	25.6
Excellent	3.01–4.00	23	14.4
Total		160	100

**Table 4:** Physical Environment Quality Distribution among ECCD Centers in Cape Coast Metropolis.

Physical Environment	Poor (%)	Fair (%)	Good (%)	Excellent (%)
Planning	68 (42.5)	89 (55.6)	3 (1.9)	0 (0)
Building as a Whole	0 (0)	89 (55.6)	45 (28.1)	26 (16.3)
Indoor Space	25 (15.6)	71 (44.4)	39 (24.4)	25 (15.6)
Outdoor Space	15 (9.4)	89 (55.6)	51 (31.9)	5 (3.1)

**Table 5:** Results of Chi-square Test and Descriptive Statistics for CenterAuspices’ Association with the Quality of Physical Environment of ECCD Centers in the Cape Coast Metropolis.

Quality of Physical Environment				
Auspices	Fair (%)	Good (%)	Excellent (%)	Total
Public	39 (66.1%)	13 (22.0%)	7 (11.9%)	59 (100.0%)
Private	50 (53.2%)	28 (29.8%)	16 (17%)	94 (100.0%)
Total	89 (58.2%)	41 (26.8%)	23 (15%)	153 (100.0%)

Note: N = 153. *χ^2^ = 2.49, df = 2. Numbers in parenthesis indicate row percentages. *p* = 0.288.

**Table 6:** Demographic Characteristics of Participants Interviewed. (HR: Head as respondent)

Participants ID	Gender	Age	Years of Experience	Qualification	Pre-School Type
HR 1, 2, 4, 5	Female	Above 35	3, 26, 8, 12	M. Phil, First Degree	Public
HR 3	Female	Under 35	3	First Degree	Private
HR 6, 7	Female	Above 35	5, 7	First Degree	Private
HR 8	Female	Under 35	12	First Degree	Public

**Table 7:** **a**: Themes Identified from the Interview on the Importance of Spaces. **b**: Extracted Responses Concerning the Themes: “Learning Outdoors”, “Upkeep”, and “All work and No Play”. (HR: Head as respondent).

**a**		
**Theme**	**Meaning**	**Number of Codes Assigned**
Learning Outdoors	Respondents gave an indication that children also learn when they are playing outdoors.	8
Upkeep	Playing outdoors contributes to the general wellbeing and upkeep of children.	7
All Work and No Play	Children need to play because they cannot be learning in the class all the time.	6
**b**		
**Participants**	**Narratives**	
**“Learning outdoors”**		
HR 4	We know that, with preschool, children learn through playing, so while they are playing they are also learning; it is very necessary.	
HR 8	It is very important for the kindergarten KG, we are doing the “SABER” program and we have indoor activities, which are table-top activities, and we have outside activities too, so they go and have some activities outside. We have to provide some things outside for them to use... aside from their playing outside, they also learn outside.	
**“Upkeep”**		
HR 7	It is very important. In most instances, they have to come out and enjoy a bit of sunshine, so providing outdoor space for them to maneuver and play helps them stretch their legs.	
HR 5	We have a lot of toys and equipment on our playground, some are like tunnels. People don’t know the use of these tunnels, but they are actually good for children who are unable to crawl. Something like a slide also helps children to be physically active.	
**“All work and no play”**		
HR 6	Yes... as the saying goes “all work and no play, makes Jack a dull boy”, so if you don’t provide outdoor space for children to play, it means you are making them work, work, work, and if they work throughout, their minds become tired.	
HR 4	When you teach them a little, there should be a little outdoor game so that their minds will rest for a while. You can’t teach them from morning to afternoon.	

**Table 8:** **a**: Themes Identified from the Interviews on Why Playgrounds do not Meet the Developmental Needs of Children. **b**: Extracted Responses Concerning the Theme on “Funds” and “Government”. (HR: Head as respondent).

**a**		
**Theme**	**Meaning**	**Number of Codes Assigned**
Funds	Challenge with getting enough equipment for children to play with; has to do with funds (money).	8
Government	The expectation is that the government should provide playing equipment for children.	4
**b**		
**Participants**	**Narratives**	
**“Funds”**		
HR 6	It is the availability of funds because when we want to get equipment for the children to play with, there seems to be no money, the capitation grants do not capture it, in fact, it is a problem.	
HR 5	If you look around, the majority of the playing equipment is destroyed but what can I do? Replacing them is quite a challenge because they are expensive.	
HR 3	With this school, we don’t have sponsors. We sponsor ourselves so we buy our teaching materials from the school fees that parents pay, some don’t pay at all and some pay it in bits. Some parents will pay half, while others won’t pay the rest so getting enough money to provide these things (playing equipment) is very difficult.	
**“Government”**		
HR 4	For the equipment, the government should supply all these materials. Once it is a government school, I think they should be able to provide all these things.	
HR 8	The office (Government Education Office) does not provide these educational materials. If we want them (equipment), we will have to use our own money to build up something for the children to use.	
